# Metal Ion Dynamic Nuclear Polarization in Mn(II)-Doped CdS Nanocrystals: Atomic-Scale Investigation of the Dopant and Its Host

**DOI:** 10.1021/acsnano.5c01257

**Published:** 2025-04-28

**Authors:** Ran Eitan Abutbul, Daniel Jardon-Alvarez, Lothar Houben, Ofra Golani, Ehud Sivan, Raanan Carmieli, Ilia Kaminker, Michal Leskes

**Affiliations:** † Department of Molecular Chemistry and Materials Science, Weizmann Institute of Science, Rehovot 761000, Israel; ‡ Department of Chemical Research Support, Weizmann Institute of Science, Rehovot 761000, Israel; § Department of Life Sciences Core Facilities, Weizmann Institute of Science, Rehovot761000, Israel; ∥ School of Chemistry, Faculty of Exact Sciences, Tel Aviv University, Tel Aviv 69978, Israel

**Keywords:** nanocrystals, doping, electron microscopy, electron paramagnetic resonance, dynamic nuclear polarization, solid-state NMR, defects

## Abstract

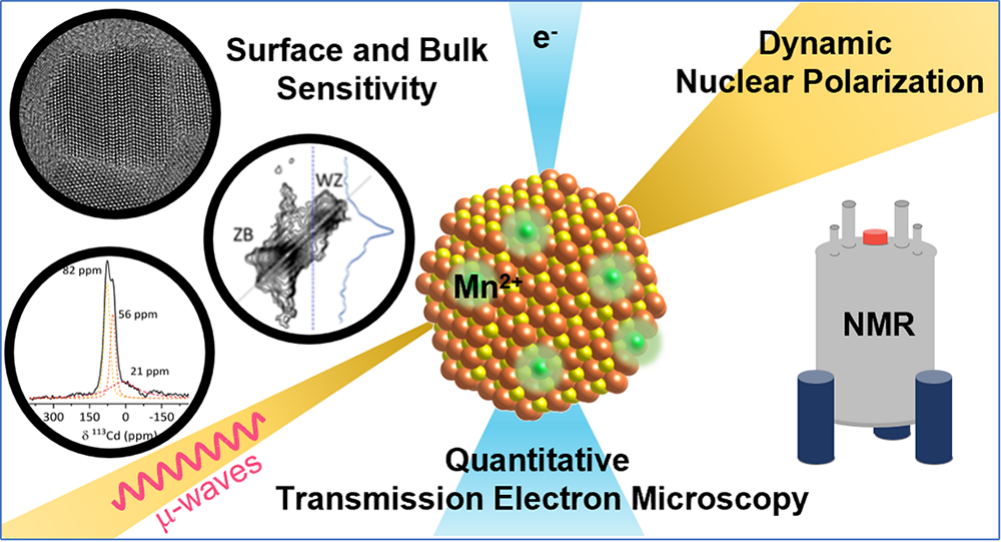

Development of functional nanocrystals requires precise control over their composition and structure. Particularly, surface composition, defects, and doping play a central role in our ability to develop functional nanomaterials. As such, there is great interest in capturing these properties. Solid-state NMR spectroscopy is a powerful tool for probing structural and compositional features at the atomic scale, in particular, when it is coupled with the high sensitivity gained by dynamic nuclear polarization (DNP). DNP enhances NMR sensitivity by transferring high electron spin polarization to the surrounding nuclear spins. This dramatically improves the signal intensity, making it a valuable tool for detecting subtle structural features. Utilizing metal ion dopants as polarization agents for DNP has been shown to be an excellent approach to increasing ssNMR sensitivity in the bulk of inorganic solids. Here, we demonstrate the implementation of this approach to nanocrystals, focusing on Mn(II)-doped CdS, where homogeneous doping is known to be challenging while being critical for the DNP process. The intricate nature of the doping was elucidated by quantitative electron microscopy and electron paramagnetic resonance spectroscopy. We confirmed that Mn(II) doping is confined to the core of the nanocrystals and that statistically dopants are homogeneously distributed within each nanocrystal. DNP from Mn(II) dopants is then shown to increase ^113^Cd NMR sensitivity by an order of magnitude, enabling distinction between core and surface environments as well as the detection of defects in the bulk of the nanocrystals. We expect that the approach can be extended to other nanocrystals, providing an efficient route for characterizing their bulk and surface properties.

## 1 Introduction

Colloidal semiconductor nanocrystals (NCs) have been widely studied for their potential use in optoelectronics, energy conversion, biological imaging, and photocatalysis.^1^ Tailoring NCs for specific applications requires precise control over their properties, namely, their composition, structure, size, and surface.^2−5^ These properties can be changed by synthetic means or unintentionally through the formation of structural defects. Solution processes are commonly employed in the synthesis of NCs, with organic ligands used to control and stabilize the formed NCs.^1,6^ The ligands can stabilize specific surface facets or NC structures and can lead to modification of the NC properties.^6−10^ Metal ion doping is another common method that can be used to tune the NC functionality. The composition, quantity, and location of the dopants can have implications on the magnetic, optical, and electronic properties of NCs.^11^

For rationally designing functional NCs, one requires an atomic-level understanding of their composition and structure. This is often extremely challenging as it requires characterizing a low population of dopants, defects, and surface species. In recent years, solid-state NMR (ssNMR) spectroscopy has been increasingly used to explore both the core and surface structures of NCs.^12−17^ In particular, an impressive level of detail could be achieved in the study of NC surfaces and interfaces through the development of high-field magic angle spinning (MAS) dynamic nuclear polarization (DNP).^18,19^ In DNP, the high polarization of electron spins is transferred to the surrounding coupled nuclear spins through microwave irradiation. This is commonly achieved by wetting or impregnating the sample with a solution containing nitroxide biradicals, freezing the sample at 100 K, and irradiating it with microwaves. This leads to polarization transfer from the exogenous radicals to the surrounding nuclear spins across the frozen solvent molecules as well as the sample. In the case of inorganic solids, such as NCs, which are made of low-abundance or low-sensitivity NMR active isotopes, the sensitivity gain is often limited to the surface and subsurface layers of the particles. Nevertheless, this approach of DNP surface enhanced NMR (DNP-SENS)^20,21^ is extremely effective, routinely resulting in >100-fold increase in NMR surface sensitivity. As such, DNP-SENS transformed the kind of information and material systems that can be probed with ssNMR. In NC research, it enables the detailed analysis of surface chemistries and the growth of core–shell structures.^12,13,15,16^

While being a very effective and general approach, exogenous DNP also has limitations in cases where it may affect the studied sample, either due to reactivity between the NCs and solvent or radicals or by changing the interaction between the ligands and NCs. Furthermore, in order to probe defects in the bulk of the NCs, the sensitivity from exogenous sources may not extend efficiently within the core of the NCs. An alternative route to gain sensitivity in the bulk as well as probe surface–ligand interactions is to utilize endogenous electron spin sources as polarizing agents. In this case, paramagnetic metal ions can be doped into the NCs and provide a source of electron spin polarization for metal ion DNP (MIDNP). This route has been demonstrated on single crystals in the early days of DNP.^22−25^ More recently, we and others have shown that metal ions with a favorable electron spin configuration (having half-filled orbital shells) can provide ample sensitivity in the bulk and buried interfaces of inorganic solids.^26−34^ In the context of NCs, this approach seems extremely appealing if the metal ion dopants can have dual functionality, endowing desired properties to the NCs and acting as polarization agents through MIDNP for increased NMR sensitivity. Here, MIDNP can potentially enable the study of defect formation in NCs, surface interactions with organic ligands, and the nature of facet stabilization and its effect on the NC growth process.

While implementing MIDNP in micron-sized solids is relatively straightforward from the synthetic point of view, doping of NCs is notoriously challenging.^35^ This is particularly so when control over the dopant oxidation state, concentration, and distribution is desired, as is the case when specific functionalities are required. Here, we tackle these challenges with the aim of expanding the application of MIDNP to NCs. We focus on Mn(II)-doped CdS NCs, which are commonly explored due to their optoelectronic and magnetic properties^11,36,37^ and prior favorable MIDNP results from Mn(II) dopants.^27,34^ We investigated CdS NCs and the dopant properties under different synthetic conditions. Utilizing electron paramagnetic resonance (EPR) with hyperfine spectroscopy, we directly determine the dopant incorporation within the CdS particles. Transmission electron microscopy (TEM) with energy-dispersive X-ray spectroscopy (EDS) and image processing algorithms all provide a detailed insight into the dopant distribution within the particles. Such insight is essential in general for understanding the dopant effect on the NC functionality, and in this study, it is important for understanding the viability of the MIDNP approach in NCs. We then examine the efficacy of Mn(II) MIDNP and utilize the approach to probe surface and bulk Cd sites and the formation of defects. Overall, we provide a comprehensive description of Mn(II)-doped CdS NCs, revealing the potential of MIDNP in the study of complex NC systems.

## 2 Results

### 2.1 Synthesis of Mn(II) doped CdS and EPR Characterization

**Figure 1:**
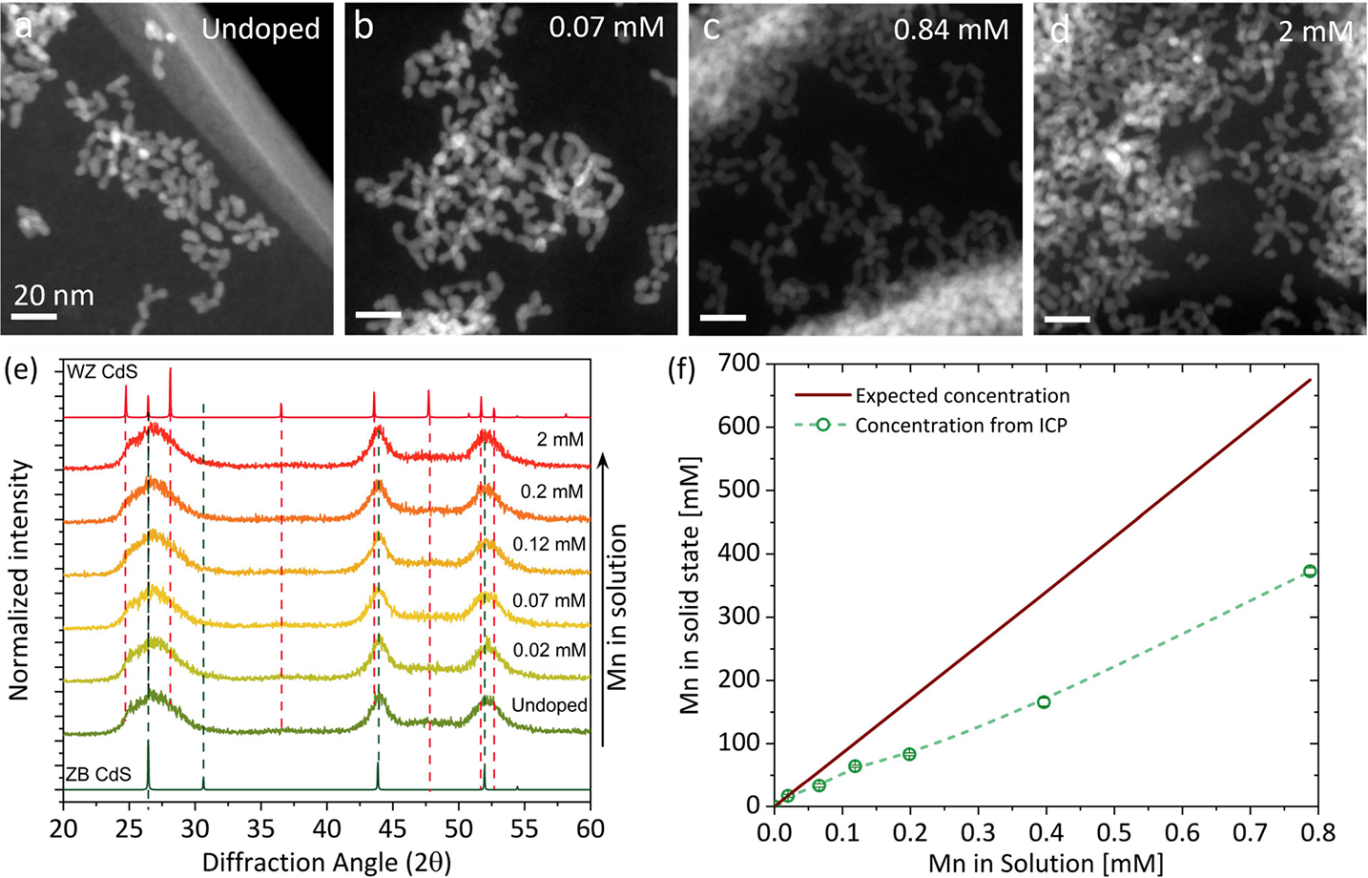
(a–d) STEM micrographs of CdS NCs with increasing concentrations of Mn (the concentration specified is for Mn in the reaction mixture). (e) XRD patterns of Mn-doped CdS NC made with increasing Mn concentrations in the reaction. (f) Measured Mn concentration in the purified nanocrystalline powders of Mn-doped CdS determined by ICP-MS (green) and the expected concentration based on the solution mixture (red).

Mn(II)-doped CdS was chosen as a suitable system for exploring MIDNP in NCs. The first step toward that goal was to confirm that we could systematically dope CdS NCs with Mn(II) without inducing any changes to the particle morphology or crystal structure. CdS NCs capped by octylamine were synthesized as described in Section 4. The Mn concentration in the reaction solution was systematically increased from 0 to 2 mM. TEM micrographs shown in Figure 1a–d reveal the morphology of the CdS NCs. In the early stages of NC growth (see Section 4), the resulting NCs exhibit an irregular morphology, often showing a tendency toward tubular shapes within the examined concentration range. X-ray diffraction (XRD) patterns obtained from each sample indicate the size of the NCs and their phase (Figure 1e). Comparison between the doped and undoped samples reveals that they are all similar in their peak shapes and intensities. The width of the obtained XRD reflections indicates nanosized particles and does not allow for a reliable quantitative analysis of the phase composition. High-resolution (S)TEM images showed that the CdS particles are not of a single crystallographic phase and contain a high density of stacking faults. This explains the partial dampening of (102) and (103) reflections of the hexagonal stacking in wurtzite (WZ) CdS.^38^ Inductively coupled plasma mass spectrometry (ICP-MS) was used to determine the Mn:Cd ratio in each synthesized sample after the purification process of the NCs. The obtained Mn/Cd ratios presented in Figure 1f are expressed as the solid-state concentration of Mn in the CdS lattice and will be used in the rest of the text to refer to different samples. Solid-state concentrations were calculated based on the ZB structure (and would not be much different for WZ as the density of the structures is similar). The red line represents the expected solid-state concentration, assuming that the Mn/Cd ratio would match that in the solution. However, while a systematic increase in the Mn concentration in the solution results in an increased Mn/Cd ratio in the purified NCs, the observed ratio (green line) is lower by a factor of ∼2 than the expected concentration. Overall, the TEM and XRD data clearly indicate that the morphology and internal structure of the NCs do not vary with an increasing Mn content in the NCs. Nevertheless, these results do not indicate whether the Mn cations are incorporated in the CdS lattice or are coordinated on the surface, interacting with the organic ligands.^35,39,40^

**Figure 2:**
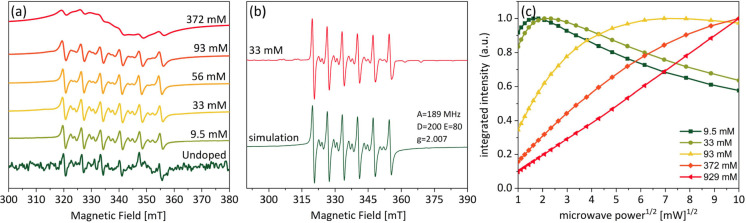
(a) Representative CW EPR spectra of the Mn-doped CdS NC. (b) EPR spectrum of Mn-doped CdS doped with 33 mM Mn (red) compared with an EasySpin^41^ simulation (green). (c) EPR saturation curves of Mn-doped CdS in various concentrations. All measurements were done on an X-band spectrometer at 100 K.

For characterizing the Mn dopants, we employed EPR spectroscopy. In Figure 2a, continuous-wave (CW) EPR spectra are shown for samples with varying Mn content. The spectrum of the undoped sample reveals the presence of a minor Mn(II) impurity, likely from the precursors used in CdS synthesis. The concentration of the Mn impurity was below the detection limit of the ICP-MS measurements. Once the Mn precursor is added in the synthesis, the EPR signal in the solid NCs increases gradually. The EPR spectra confirm the presence of Mn(II) (electron spin *S* = 5/2) with the characteristic sextet lines due to hyperfine couplings with the ^55^Mn nuclear spin (also spin 5/2). Above 100 mM, the spectra start to broaden due to the increased electron interactions and a decrease in the electron relaxation times. In Figure 2b, the CW EPR spectrum of the 33 mM Mn-doped CdS sample is compared with a simulation performed in EasySpin.^41^ The simulation allows us to determine the g factor as 2.007 with a hyperfine constant to ^55^Mn nuclei of 189 MHz. This value is consistent with that of a covalently bonded Mn(II) ion in II–VI compounds at a tetrahedral substitutional position.^42^ Insight into the relaxation properties of the electron spins was obtained from CW microwave saturation curves (Figure 2c). The required microwave power to reach a maximum in the intensity of the Mn(II) resonances increases with increasing Mn(II) content. The saturation curves were fitted using the steady-state solution to the Bloch equation,^43,44^ which provides an estimation of the longitudinal Mn(II) relaxation times. These were estimated to vary from 3 to 0.02 μs for Mn(II) in the concentration range of 9.5–372 mM, assuming equal longitudinal and transverse electron relaxation (Table S1). The decrease in relaxation times, observed with increasing Mn content, can again be attributed to an increase in the Mn interactions in the samples.

**Figure 3:**
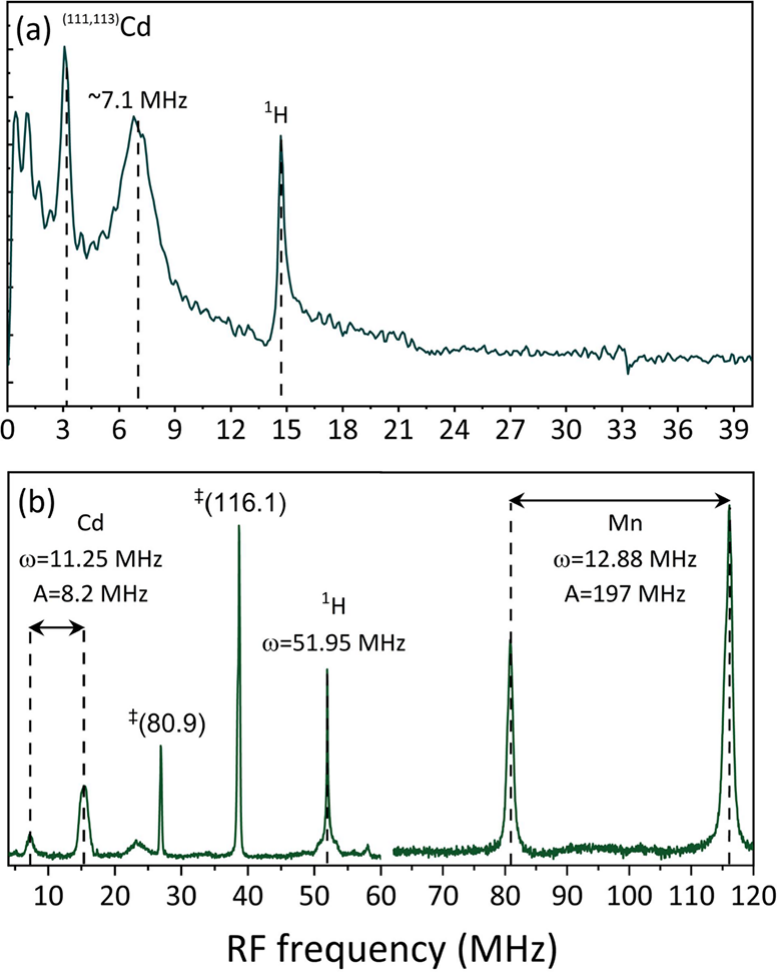
Hyperfine spectroscopy measurement performed on 93 mM Mn-doped CdS synthesized with 10 min incubation time. (a) ESEEM spectrum acquired on an X-band system at 15 K and (b) ENDOR spectra acquired on a Q-band at 15 K. The assignment of each line is denoted on the spectrum. The label ‡ refers to the third harmonics of the frequency specified in the brackets.

Further insight into the Mn position in the NCs was obtained from electron spin echo envelope modulation^45,46^ (ESEEM) experiments. In ESEEM, the modulations in the electron spin echo as a function of echo time, arising due to the electron couplings with the surrounding nuclear spins, are measured. Applying a Fourier transform to the time domain trace reveals a spectrum of the nuclear Larmor frequencies and hyperfine interactions of coupled nuclei. Figure 3a shows the ESEEM spectra of the 17 mM Mn-doped CdS NCs. Three lines are observed at different frequencies: 3.1, 7.1, and 14.7 MHz. Given that the ESEEM experiment was carried out at a magnetic field of 0.35 T (X-band), the Larmor frequencies of ^111^Cd, ^113^Cd, and ^1^H are 3.16, 3.3, and 14.9 MHz, respectively. Hence, the line at 3.1 MHz is assigned to both Cd isotopes, and the line at 14.7 MHz is assigned to ^1^H. The line at 7.1 MHz is assigned to Cd nuclei with strong hyperfine couplings to Mn(II) electrons, stronger than the nuclear Larmor frequency. This line is shifted by 4.1 MHz from the Cd Larmor frequency, suggesting the presence of an Mn(II)–Cd hyperfine interaction of 8.2 MHz (a second line, which is expected close to 0 MHz, cannot be resolved clearly in the ESEEM spectrum). Considering that the shortest Cd–Cd distance in the lattice is approximately 4 Å and assuming Mn replaces Cd within the lattice, we would expect the strongest through-space dipolar coupling to be on the order of 0.25 MHz. The large coupling of 8.2 MHz is therefore assigned to isotropic Fermi contact interaction between the dopant electrons and Cd, which would only be measurable if Mn(II) ions are incorporated within the CdS lattice. The resonance at 7.1 MHz is also significantly broadened by the Cd–Mn(II) through space dipolar interactions (as well as the presence of two Cd isotopes). The ESEEM results show the presence of two types of Cd populations: one distant, weakly coupled to the electrons and a second population of Cd nuclei in close proximity to Mn(II) with strong coupling to its electrons. These results provide strong evidence that Mn(II) was successfully incorporated into the CdS lattice.

Additional confirmation for the successful doping was obtained from an electron nuclear double resonance^47−49^ (ENDOR) experiment (Figure 3b). The ENDOR spectrum was recorded at a higher magnetic field (Q-band), where the Cd Larmor frequency is larger than the hyperfine couplings. This allows us to clearly identify the pattern arising from Mn(II) surrounded by Cd nuclei, with two lines at 7.1 and 15.35 MHz separated by 8.2 MHz as a result of the isotropic hyperfine interaction and centered around the Cd Larmor frequency at 11.25 MHz. The additional lines at 80.8 and 116 MHz arise from the ^55^Mn nucleus (centered at roughly half the hyperfine coupling and split by roughly twice the ^55^Mn Larmor frequency). The signals at 27 and 39 MHz are 3rd harmonic lines of ^55^Mn (an experimental artifact). The line centered at 52 MHz corresponds to the Larmor frequency of ^1^H. The sharp components of this line arise from distant, weakly dipolar coupled ^1^H nuclei. The broad component arises from ^1^H nuclei that are strongly coupled to Mn(II).

Overall, our EPR results confirm the incorporation of an increasing amount of Mn(II) in the solid with most Mn(II) dopants in a symmetric environment, likely substituting Cd sites in the core of the NCs as indicated by the narrow CW EPR lines and the strong isotropic hyperfine coupling to Cd nuclei. A low amount of Mn(II) dopants has strong coupling to ^1^H nuclei in the ligands, suggesting a minor contribution of surface doping. The position of the dopant in the NC is important for the function of the particles as well as for the utilization of Mn(II) in DNP. Quantitative insight into the distribution of Mn(II) dopants in the particles is obtained from TEM studies.

### 2.2 Dopant Distribution from Quantitative TEM

**Figure 4:**
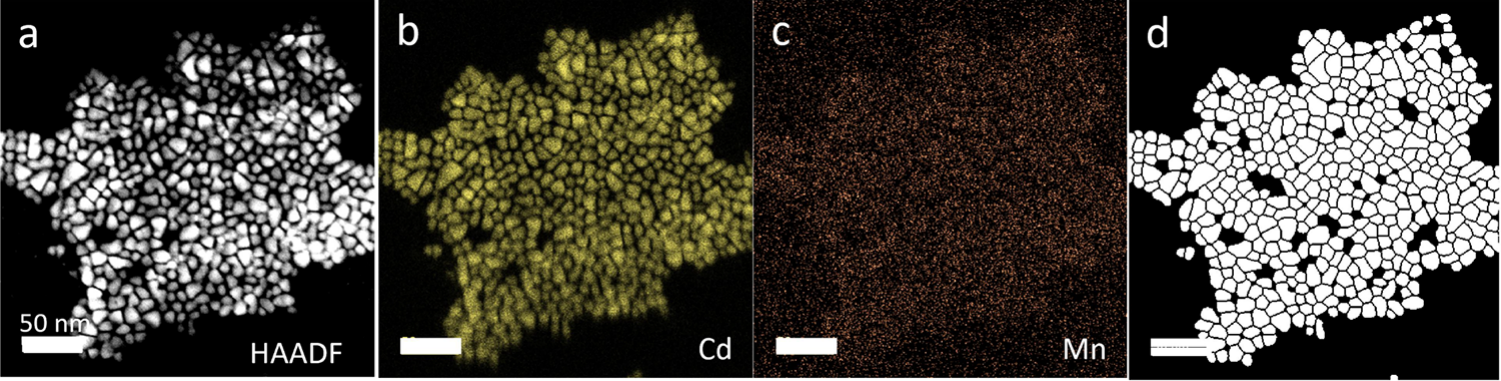
STEM data used for quantitative electron microscopy on a CdS sample incubated for 24 h in a solution containing 0.2 mM Mn(II). (a) STEM micrograph of Mn-doped CdS NCs and corresponding elemental maps of (b) Cd and (c) Mn. (d) Segmentation of the image in (a) to particle boundaries using Cellpose 2.

The distribution, concentration, and position of Mn dopants in the CdS NCs are expected to influence their EPR properties and, subsequently, the resulting DNP performance from the samples. The macro-scale properties of the doped samples will exhibit features that arise from a collective of NCs, which may differ in size, shape, amount of doping atoms, and their location within a single NC (surface or core).^50^ For characterizing the dopants in the nanoscale, we performed a detailed investigation using high-resolution scanning TEM (HR-STEM) equipped with EDS. Figure 4 depicts the STEM micrograph, the corresponding background-subtracted elemental maps of Cd and Mn, and segmentation of the image into individual particles. Mn is clearly detected (Figure 4c), but the naturally low X-ray emission cross sections together with the low amount of Mn inevitably result in low signal-to-noise ratio data that do not allow the direct localization of Mn.

For a quantitative analysis of the Mn distribution across CdS particles, the data were collected from many NCs. The images were then processed using image analysis software, providing a statistical view of the acquired data with reference to an average particle and the most probable location of Mn atoms. We first describe this process by simulations of different scenarios of dopant distributions within the particles, followed by comparison with the analysis of the experimental data.

In STEM EDS at a length scale larger than the atomic scale, the observed elemental map approximates a 2D projection of a 3D object. This means that features in the projected elemental maps cannot be directly assigned to the surface or the core of the object. However, in the limit of a large number of X-ray counts, assignments can be made by examining, for example, the signal obtained from the 2D projection of a solid sphere vs its shell. For a solid sphere, the expected 2D projection would include high intensities around the center of the projection and lower intensities toward the edges of the object. However, for a shell, equally low intensities are expected for most of the area of the object except for its edges, where the intensity should be higher. This difference in projections allows us to distinguish between the two different doping scenarios of core vs. surface.

The element-density-projection-simulated images are designed to represent Cd and Mn elemental maps under two extreme scenarios in order to validate that the two doping cases are distinguishable. To test whether this method can differentiate between random Mn occupancy and Mn surface segregation, we generated simulated input files with known parameters. This approach is later applied to the real dataset to infer the actual Mn distribution in CdS NCs. MATLAB was used to construct 3D matrices representing spheres with size distributions according to parameters measured in STEM. In the 3D matrix, each cell corresponds to a pixel in the experimental EDS images. The average number of Cd atoms in each voxel was determined based on the assumption of a ZB structure, which inherently assumes random site occupation. The elements in the matrix were assigned accordingly. The projection of the 3D matrix, performed by summing over the third dimension, results in a simulation of the element-density projection visible in the Cd elemental maps. Assuming that only Cd and S constitute the lattice elements, the simulation results in a gradient of intensity that peaks at the center of the sphere (Figure S6a). The simulation of Cd atoms on the surface only was performed by applying the 3D Sobel operator, which is used in image processing for edge detection. Collapsing the resulting 3D matrix into a 2D image results in an image of circles with almost equal intensity in their area (Figure S6b). The simulation of the Mn maps was conducted similarly. In this case, the Cd 3D matrices were used, and each value in the 3D matrix was replaced with another value corresponding to the number of Mn atoms. The doping level was set at 1:50 (Mn:Cd) and used as the probability to replace the Cd atoms with Mn atoms. Using the Cd 3D matrices (core and surface) as a precursor for the Mn 3D matrices ensures that the substitution of Cd with Mn is carried out in the correct positions and not subjected to errors due to incorrect edge detection in the Mn map that would appear noisy after the substitution. To represent the random occupancy of Mn dopants in the NCs, we calculated how many Mn atoms could replace Cd atoms in each voxel of the matrix. The assigned value in each voxel is proportional to the fraction of Cd atoms replaced by Mn, ranging from 0 (no Mn substitution) to a maximum determined by the available Cd sites. Since we normalized the number of Cd atoms to 1 per voxel, the values in the Mn maps were adjusted accordingly to reflect this proportional replacement. The simulated elemental maps of Mn doping in the core or surface scenarios are shown in Figure S6c,d. The noise level is expected for the low substitution ratio of 1:50 (Mn:Cd).

The simulated EDS elemental maps of Mn and Cd were then analyzed by using image processing software. The first step of the analysis was to identify the boundaries of each nanoparticle. For this, particle segmentation was conducted using Cellpose 2.51,^51^ an image processing software that utilizes machine-learning techniques for image segmentation. Initially, the software was trained to identify the boundaries of the NCs until a satisfactory result was obtained. Following segmentation, the simulated elemental maps were correlated with the segmentation map, and for each NC, the signal intensity was integrated versus its distance from the particle edge. The integrated intensity obtained from each NC was then averaged across many NCs of the same size. We assume that by averaging the signal intensities we eliminate the effect of particle orientation with respect to the beam. Furthermore, by collecting data from multiple NCs, we overcome the sparseness expected in the data of the dopants since each NC contains a small number of dopants.

**Figure 5:**
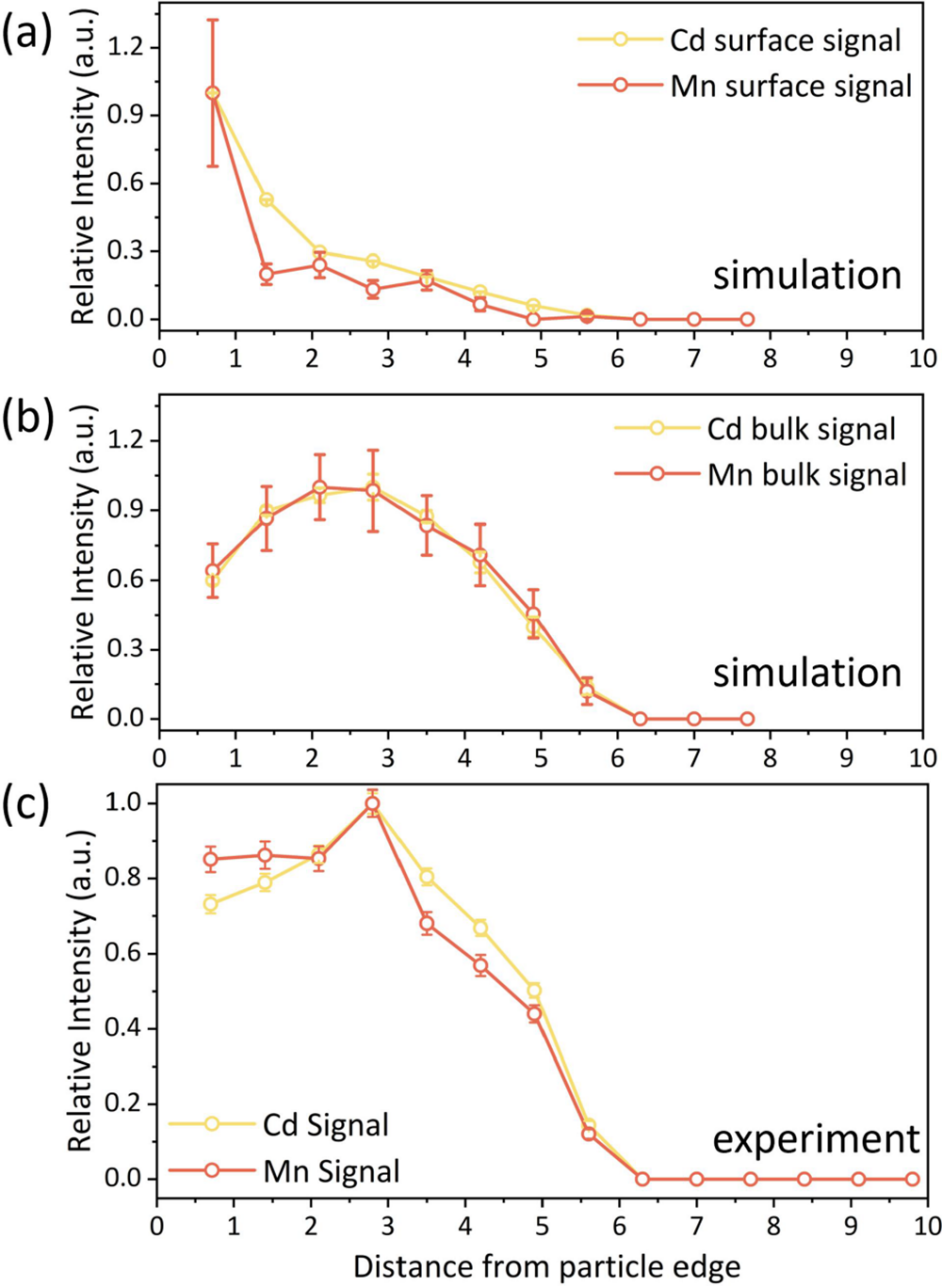
Cumulative radial integrated signal for particles with a radius of 5.6 nm: simulated data show that matrix elements and dopants are dispersed in the NC (a) surface and (b) surface and bulk with a dopant-to-matrix ratio of 1/1000. (c) Experimental curve of the radial integrated signal for particles with a radius of 5.6 nm. The integrated signal intensities were measured from both the Cd and Mn elemental maps.

The simulated elemental distribution curves are shown in Figure 5a,b, where we examined the case of random distribution of Mn and Cd in the core vs surface of the particles. Core doping results in maximal signal intensity at a specific distance from the edge of the particles, which arises due to the balance between the number of pixels (higher at the edge) and the signal intensity (lower at the edge and increases toward the particle center). In the other extreme case of surface doping, the signal intensities do not vary much across the 2D projection with maximal intensity at the edges, which in this case contain the highest number of pixels as well as signal intensity. The results of this analysis confirm that the two extreme doping schemes can be distinguished with this approach.

The experimental elemental maps were analyzed in the same way as the simulated data to extract the compositional profiles for an average particle and thus overcome the severe signal-to-noise limitation for a measurement on a single particle. It is important to note that the preprocessing for the elemental maps included the correction of the background signal, so only the net signal is accounted for in the maps. As the noise level in the resulting Mn maps was high and in order to ensure the software integrated only valid Mn signals, we only included Mn signals higher than a threshold value that was set as the noise level in the analysis. The resulting Cd and Mn signal intensities vs. distance from the particle’s edge are shown in Figure 5c. The intensity of Mn follows the trend of the Cd signal, suggesting that Mn atoms are distributed similarly to Cd—in the core of the NCs. This is validated by comparison with the curves calculated from the elemental maps simulated with a particle size of 5.6 nm (which was chosen as it was abundant in the synthesized particles). The experimental curves have a higher resemblance to the case of core doping, with the maximal intensity away from the edge. Thus, we conclude that for our synthesis conditions Mn dopants are evenly mixed in the core of the CdS nanoparticles.

**Figure 6:**
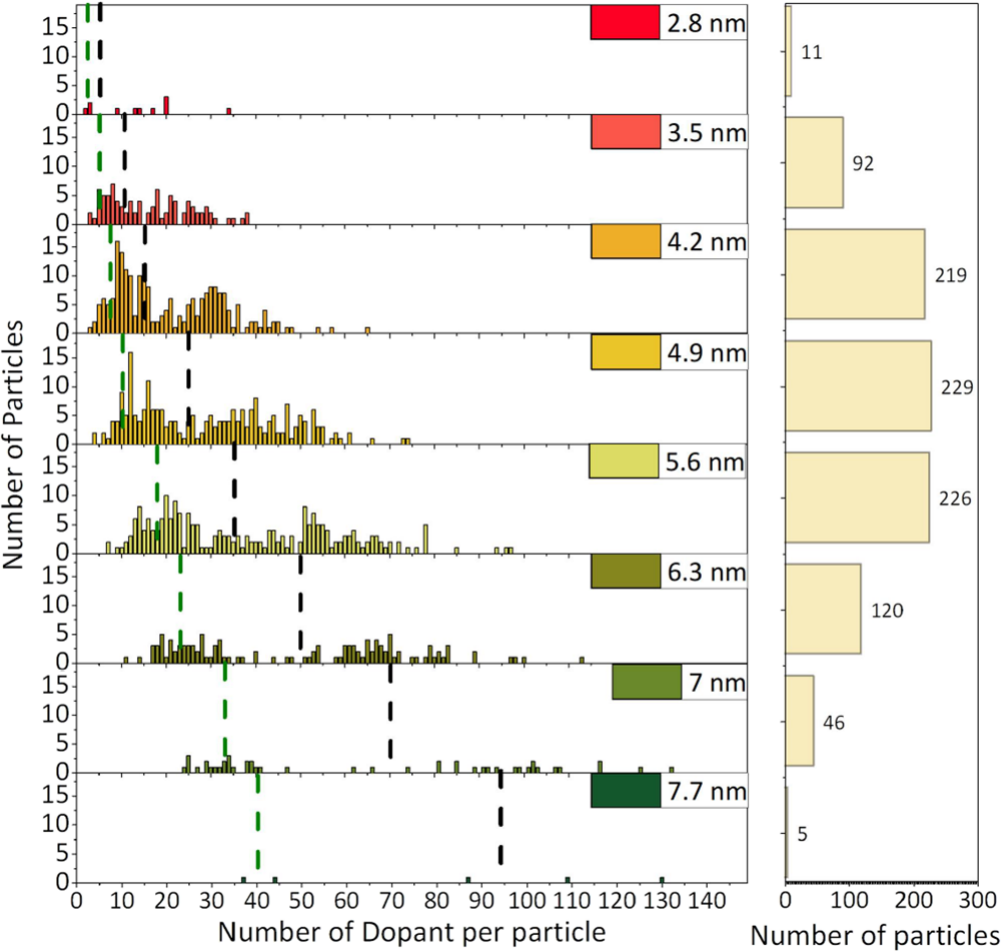
Distribution of particles as a function of the number of dopants per particle for each particle size. The histogram of particle size is shown on the right. Data are constructed from the STEM dataset for particles grown in 0.2 mM Mn in solution for 24 h. The black dashed lines are the average dopants per particle based on the corresponding ICP-MS results. The green dashed lines represent optimal dopant concentrations in bulk materials according to previous studies.

The direct measurement of Mn in CdS NCs allows us to quantify the number of dopants per NC. This was done by overlaying the elemental maps onto the segmented images, which enables assigning each Mn atom to a specific NC. From this we can determine the number of dopants present in each NC. Our nanoparticles have a relatively large size distribution, with each particle containing different amounts of Cd and Mn atoms. This distribution is presented in Figure 6, with a histogram of the number of particles per particle size with a specific number of dopants. We excluded very small or large particles from this analysis due to insufficient statistics. The black dashed lines represent the calculated number of dopants for the particular particle size, based on the concentration from ICP-MS. Comparing the black dashed line for different particle sizes shows that it consistently represents the average value of the doping level. This is a good indication that our ICP-MS and quantitative STEM approaches are valid, reporting similar doping levels. The green dashed line represents a dopant concentration of 33 mM, which was found effective for MIDNP.^27,33^ The benefit of probing the Mn doping in this approach is observing the distribution at the nanoscale, which is impossible with macro-scale methods. The dopant distribution per NC size is expected to follow Poisson statistics. However, our analysis reveals a deviation from this expectation, suggesting that additional factors influence dopant incorporation. The average value of dopants increases with an increasing size of the particles. Also, the distribution broadens for bigger particles, resulting in a larger variation in the doping level for larger particles. Interestingly, for each particle size, the distribution is centered around two distinct mean values. The mean values could be related to different doping pathways of the NC but were not investigated further in this work.

### 2.3 The Effect of Reaction Parameters on Mn Incorporation

**Figure 7:**
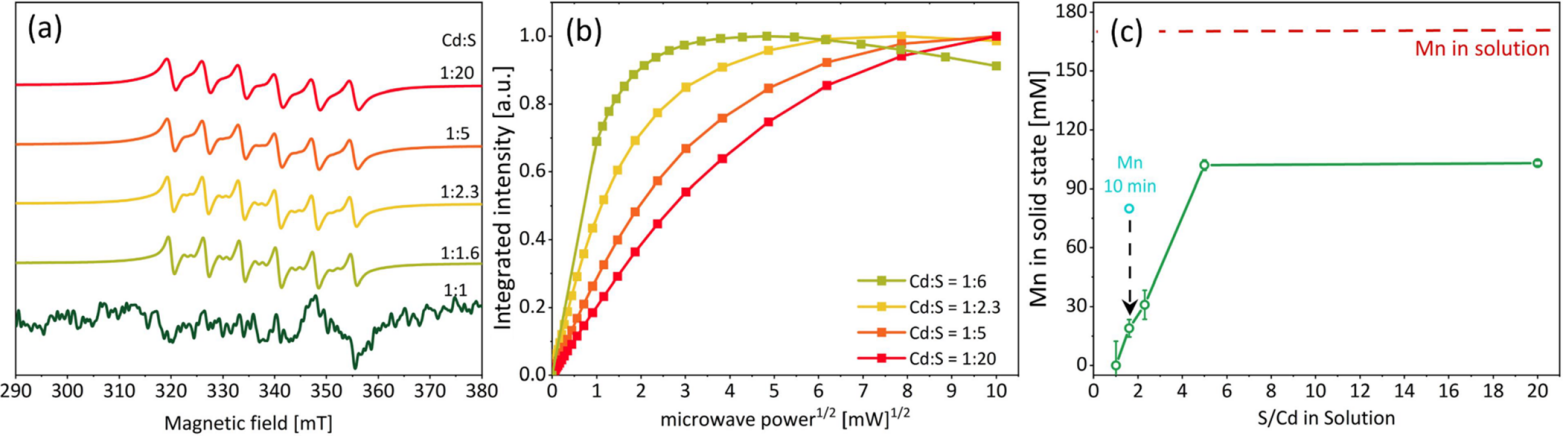
(a) CW X-band EPR spectra of CdS NCs synthesized with a constant Mn concentration in solution (0.2 mM, 24 h) with a different Cd/S ratio. (b) Corresponding saturation curves for the doped samples. (c) Mn concentrations in the solid particles obtained by ICP-MS for particles grown for 24 h (green) compared with the expected solid concentration based on the solution (red) and with a sample incubated for 10 min (cyan).

Modifying the Cd/S ratio in the reaction mixture while keeping the Mn concentration constant had a noticeable effect on the EPR spectra of the resulting NCs. In Figure 7a,b, the CW EPR spectra and saturation curves are shown for Mn-doped CdS made with increasing S content. With a decreasing Cd:S ratio, the EPR resonances associated with the Mn(II) unpaired electrons broaden (Figure 7a). Additionally, these lines require a higher microwave power to reach saturation, indicating an increase in the electron spin relaxation rates (Figure 7b). This trend suggests that despite the constant Mn content in the solution its amount increases in the solid phase. Interestingly, with a 1:1 ratio of Cd/S, there was hardly any Mn(II) incorporation in the particles. ICP-MS was used to evaluate the effect of the S content on Mn doping efficacy (Figure 7c). Indeed, for a Cd:S ratio of 1:1, the level of Mn is below the detection limit of the instrument. The increased S/Cd ratio results in an increased Mn content up to a ratio of 5, after which the variation in the incorporated Mn is minimal. As we observed before, the Mn/Cd ratio in the nanocrystalline powder is not equal to that in the solution, indicating that not all of the available Mn was incorporated in the NCs. In another experiment conducted using similar initial conditions but with a shorter incubation time, higher Mn/Cd levels were observed (Figure 7c). This indicates that with a longer incubation time the dopants are expelled from the NCs. These processes have been previously discussed in the literature, and this result aligns well with other reports.^52,53^

The effect of the S/Cd ratio on the reaction products is also manifested in the size and morphology of the NCs. In Figure S1, the STEM micrographs of samples synthesized with different ratios of precursors are shown. With a higher S content, the size and size distribution of the NCs increase. In various experiments performed with a constant S content but varying Mn contents, we did not observe changes in the particle size. Thus, we attribute the change to the effect of S content on the surface chemistry and the growth mechanism. It was shown that use of elemental S in an alkylamine solution resulted in the formation of charged species, primarily alkylammonium-polysulfides, at low temperatures.^54^ This caused dramatic changes to reaction products, depending on the researched system. In addition, at higher temperatures, the sulfur-amine solution can result in more reactive by-products that facilitate faster reaction kinetics.^54^ We suspect that the introduction of more S accelerated the reaction, resulting in larger NCs and different morphologies.

### 2.4 Metal Ion DNP in Mn-Doped CdS Nanoparticles

**Figure 8:**
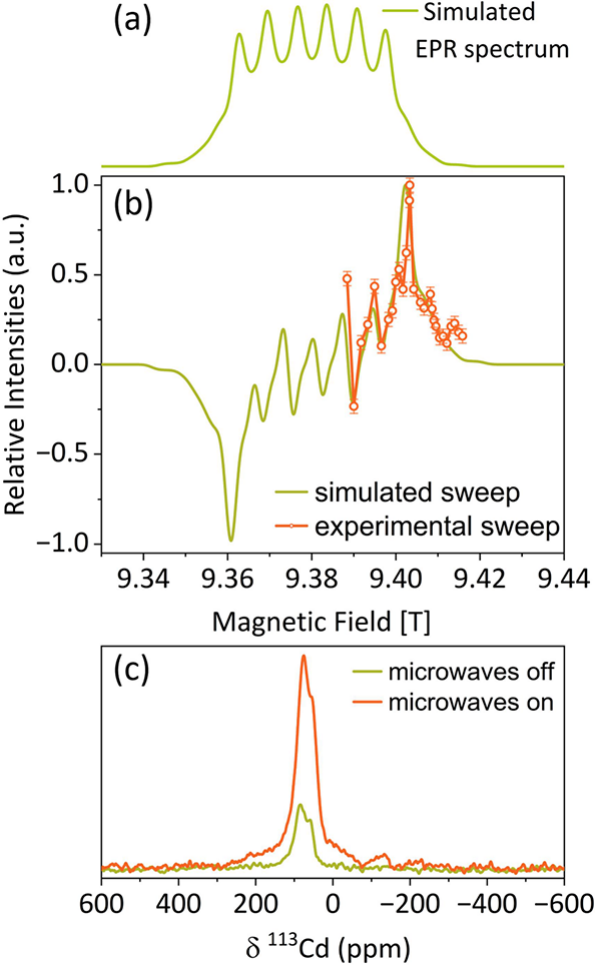
(a) EasySpin EPR simulation of the Mn(II) spectrum at the J-band (263 GHz) with ZFS and g anisotropy extracted from the low-field measurement. (b) Prediction of the solid effect DNP field sweep profile^71^ based on the EPR spectrum (green) and the experimental result (orange) obtained for 83 mM Mn-doped CdS. (c) Corresponding ^113^Cd direct excitation NMR spectrum of Mn-doped CdS NCs obtained with (orange) and without (green) microwaves.

As we established that Mn(II) ions are predominantly incorporated within the NC, we now turn to explore their viability as polarization agents in MIDNP. First, it is important to determine the optimal field position for performing the DNP experiments. The EPR transitions and nuclear Larmor frequency determine the optimal position for DNP. A simulation of the Mn(II) EPR spectrum at high field (9.4 T used for DNP-NMR experiments) was performed based on the low-field measurement. It was then used to phenomenologically simulate the expected field sweep profile (Figure 8a,b).^55^ The most common mechanism in MIDNP is the solid effect,^33^ where we expect DNP positive and negative enhancement at the sum and difference of the electron and nuclear Larmor frequencies, respectively. In the case of Mn(II), each of the hyperfine lines can give rise to positive and negative enhancement, but since the Mn hyperfine constant (*A* = 189 MHz) is approximately equal to double the ^113^Cd Larmor frequency (ω_*n*_ = 88.72 MHz), it results in the partial cancellation of adjacent lobes. Hence, the optimal condition for ^113^Cd nucleus DNP enhancement is expected to occur at the highest or lowest field positions, where there is no cancellation. Indeed, the sweep profile measured on 83 mM Mn(II)-doped CdS NC (acquired for half of the range) followed the expected field dependence. For all other measurements, the field was set to the position of the highest positive enhancement, resulting in the ^113^Cd spectrum shown in Figure 8c.

**Figure 9:**
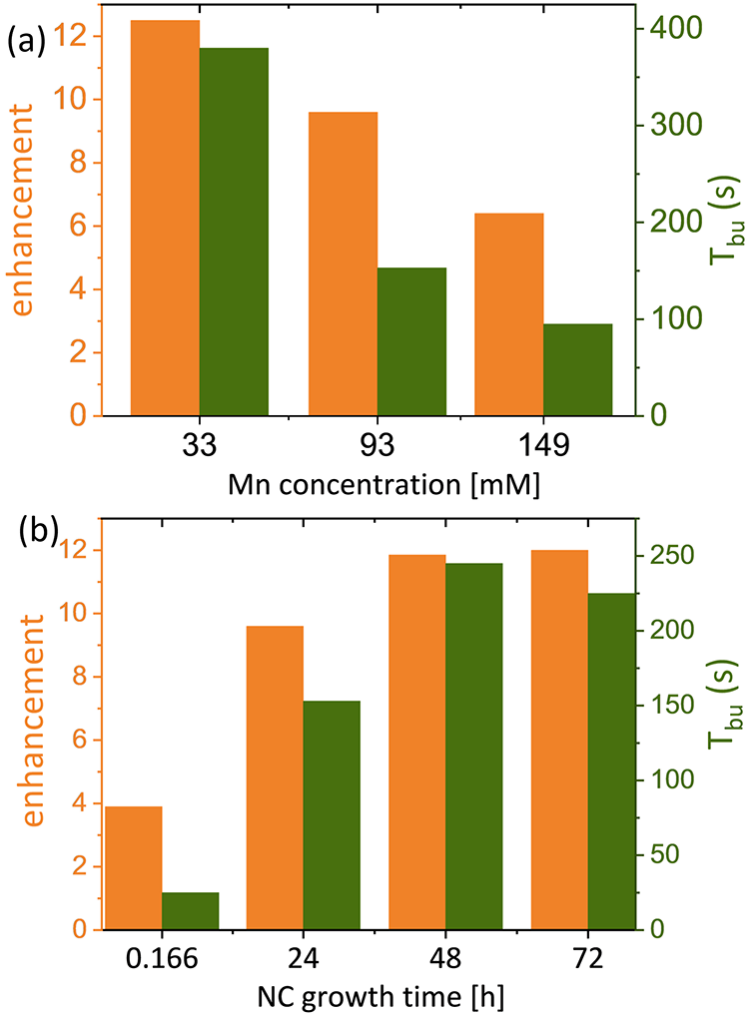
DNP enhancement factors (orange) for ^113^Cd and DNP polarization times (green) determined as a function of the (a) Mn(II) concentration and (b) incubation time.

We first examined the effect of reaction time and dopant concentration on signal enhancement. For each of the samples, we measured the DNP build-up time, *T*_bu_, and the enhancement factor defined as the ratio between the spectral intensity with and without microwave acquired at steady state, marked as ε_on/off_ (Figure 9). Examining first the effect of Mn(II) concentration (Figure 9a), we observed that the polarization time and the DNP enhancement decrease with Mn(II) content in the samples. This can be attributed mostly to the decrease in electron relaxation with Mn(II) content (Figure 2c), which makes it harder to saturate the electron spin transition and results in lower DNP efficacy. Overall, the enhancement factors observed are relatively low, especially considering the relatively favorable relaxation properties of Mn(II) in these particles. These can be understood considering two factors—the broad distribution of dopants in the NCs, with many particles being undoped (and thus not contributing to the DNP process), and the presence of inherent nuclear relaxation sources for ^113^Cd nuclei in the NCs. We expect that the presence of defects and protonated ligands in the samples would provide an efficient route for nuclear relaxation, which would limit the range of polarization transfer from the Mn(II) dopants.^34^ The electronic properties of the NCs can also be an efficient source of relaxation as well as cause sample heating under microwave, which was shown to significantly lower the DNP performance.^56^ The decrease in polarization time can be attributed to more ^113^Cd nuclei being coupled to Mn(II) dopants in the NC, which results in shortening of the nuclear relaxation time. Examining the STEM micrographs (Figure S2) excludes the effect of size and shape since all analyzed samples did not differ much in size and distribution. Next, we examined the effect of incubation time, which is usually associated with particle growth, ripening processes, and defect annihilation. The temporal evolution of the NCs (Figure S3) indicates significant particle growth, mainly during the first 24 h of the reaction. In addition to a considerable increase in the average size, the shape of the particles becomes distinctively tetrahedral.

The polarization time and enhancement obtained as a function of NC growth time are plotted in Figure 9b, showing an increase in both parameters with the incubation time. This can be explained by the increasing NC size and degree of order in the particles. Both of these would lead to an increase in electron and nuclear relaxation times, which would improve the DNP performance. Increasing the incubation time also results in a loss of dopant over time. This effect was discussed earlier (Figure 7c) and by others,^52,57^ and here, it would result in reduced Mn–Mn interactions and, therefore, reduced electron relaxation rates. Table S3 summarizes the Mn(II) relaxation times, showing these increase from incubation of 10–48 h and then decrease at 72 h, which partially aligns with the observed behavior of the enhancement factors.

### 2.5 Detecting NC Bulk, Defects, and Surface with MIDNP

**Figure 10:**
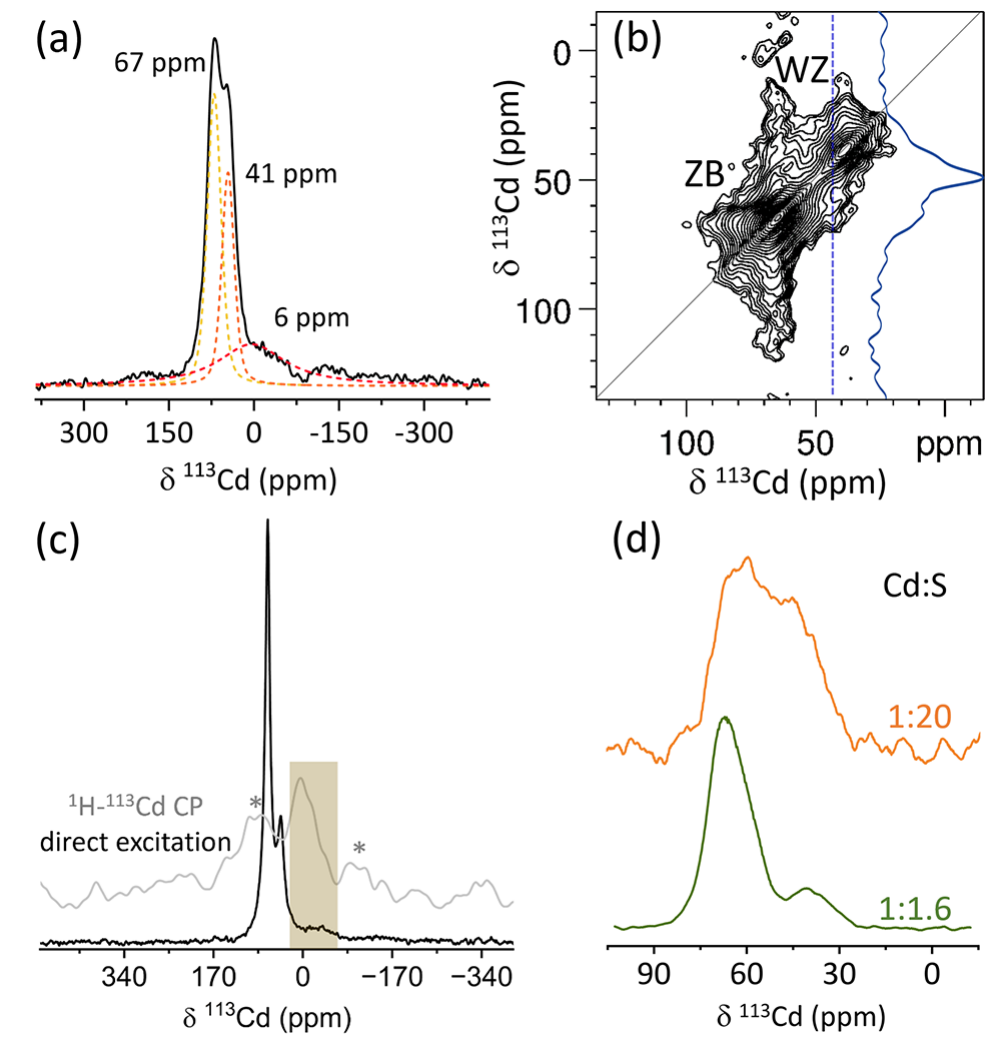
(a) ^113^Cd DNP enhanced spectrum of 19 mM Mn-doped CdS NCs (10 min incubation time) and deconvolution to components. (b) DNP enhanced ^113^Cd–^113^Cd RFDR homonuclear correlation spectrum obtained with 55 ms mixing time for CdS incubated for 24 h in a solution containing 0.2 mM Mn. The blue spectrum is a vertical slice taken at 41.3 ppm showing the correlation between different environments. (c) ^113^Cd NMR spectra of 169 mM Mn(II)-doped CdS particles grown for 72 h. The spectrum in black was acquired with direct excitation with microwave on and is compared to a ^1^H–^113^Cd cross-polarization (CP, gray) acquired with 1 ms contact time and no microwave irradiation. Spectra were acquired with 9 kHz MAS; spinning side bands are marked with asterisks. The highlighted region is assigned to surface Cd sites. (d) DNP enhanced ^113^Cd spectra extracted from the diagonal of RFDR spectra of CdS incubated for 24 h in a solution of 0.2 mM Mn with different ratios of Cd:S.

We now turn to examine the Cd environments detected by MIDNP. The ^113^Cd spectrum shown in Figure 10a shows three distinct chemical environments, intense resonances at 67 and 41 ppm, and a broader resonance at 6 ppm. We attribute the sharp and broad resonances to core and surface environments, respectively. In our previous work, we found that in crystalline solids where the paramagnetic dopant is the main source of nuclear relaxation the polarization is uniform across the lattice (and thus essentially independent of the distance from the dopant). The decrease in *T*_bu_ with increasing Mn(II) content suggests Mn(II) is a major source of relaxation in these samples as well, yet due to the disordered nature of the particles, we cannot rule out other sources of nuclear relaxation. However, in the previous sections, we established the uniform core doping of Mn(II) in the NCs. As such, we can expect, on average, a close to uniform enhancement of the different environments across the ensemble of NCs (although not all Cd nuclei will be polarized as some particles are not doped at all). This allows us to estimate the comparison of the distribution of sites from the DNP enhanced spectra. Deconvolution of the spectrum with DMFIT^58^ software reveals a ratio of 1.4:1 between the 67 and 41 resonances. The XRD diffraction pattern for this sample and the electron microscopy images suggest that the formed particles are highly defective structures, displaying both cubic and hexagonal short-range order. As ssNMR is a sensitive probe for the local environment of ^113^Cd (first coordination shell and beyond it), it can be used to determine the extent of these two main ordering schemes. As bulk CdS with a ZB structure resonates at 65 ppm^59,60^ (Figures S4 and S5), we assign the sharp resonance at 67 ppm to a ZB-type environment. The second bulk environment at 41 ppm is assigned to Cd sites in the WZ-type hexagonal ordering based on its lower resonance frequency^61^ (also observed comparing CdSe with ZB and WZ ordering^60^). The broadening of the resonance at 6 ppm suggests that it arises from surface sites. This assignment was also supported by ^1^H–^113^Cd cross-polarization, where ^1^H polarization in the ligands is transferred to nearby ^113^Cd sites (Figure 10c). The broad resonance detected and centered at roughly 6 ppm could be a consequence of the low site symmetry or reflect the heterogeneity of the surface. Octylamine, the solvent used during NC synthesis, is known to be a weak ligand for CdS NCs^62,63^ binding through the amine head group. Therefore, we assign this resonance to surface-bound Cd in the vicinity of an amine.

The increased sensitivity of MIDNP to core sites can be leveraged to investigate the local structure of Cd sites in the NCs. This can be achieved through correlation experiments that reveal the spatial proximity between Cd sites. If present, a correlation between WZ and ZB-type Cd sites would indicate their spatial proximity. To examine the spatial proximity between the Cd local environments, we recorded a ^113^Cd–^113^Cd homonuclear correlation experiment. We have utilized radio-frequency-driven recoupling^64,65^ (RFDR) to reintroduce the distance-dependent dipolar interactions, which are averaged to some extent by spinning the sample. The 2D correlation map (Figure 10b) is expected to show each Cd site along the main diagonal. A correlation between sites would appear as an off-diagonal cross-peak, indicating spatial proximity (a few Angstroms) between the corresponding Cd sites. The main diagonal peaks can be assigned to cubic and hexagonal Cd environments. Additionally, the diagonal pattern reveals minor signals at intermediate chemical shifts, suggesting additional Cd environments. In Figure 10b, weak off-diagonal cross-peaks can be observed between ∼60 and ∼45 ppm, indicating these environments (which are likely very structurally similar to the main ZB and WZ coordination) are formed in close proximity in the crystal. This observation is consistent with a structurally heterogeneous system, possibly due to interface mixing between ZB and WZ domains or local disorder near the interface. To explore the origin of these additional resonances that are not assigned to either pure cubic or hexagonal local structures, additional experiments were performed on samples varying in the Cd:S ratio in the reaction mixture (Figure 10d). With increased S content, we observed an increase in the number of Cd resonances (here, extracted from the diagonal of the 2D experiment), spanning the frequency range between the two main cubic and hexagonal environments and reflecting a distribution of chemical environments in the NCs (Figure 10d), as well as some weak correlations between them.

**Figure 11:**
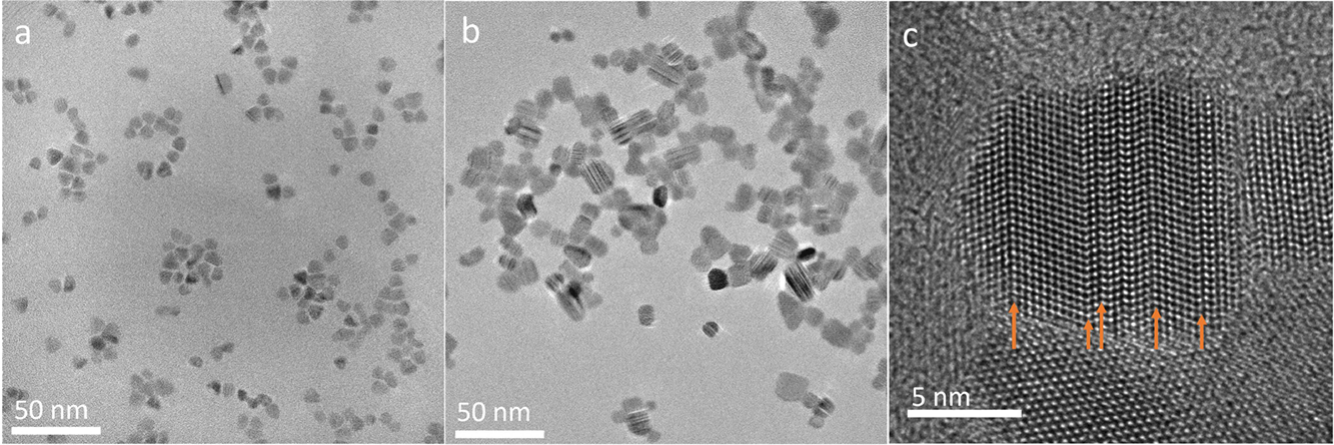
Bright-field TEM micrographs of samples synthesized with (a) 1:1.6 and (b) 1:20 Cd:S ratios. (c) HR-TEM of CdS particles containing multiple stacking faults (with a 1:20 Cd:S ratio).

To further examine the source of these new environments, we acquired bright-field TEM images of the two samples (Figure 11a–c). In the two samples, we observe stacking faults by their characteristic Bragg contrast.^66,67^ We observed a higher density of stacking faults with increasing Cd:S ratios. As TEM does not reflect the entire sample composition but only the examined region, we also performed a careful XRD analysis of the samples prepared with different S contents (Figure S7). Despite the significant broadening in all samples expected from their nanoscale order, we identified a more pronounced broadening of the ZB (200) reflection at 30.7° with increasing S content in the synthesis. Examining the characteristic particle size of low and high S contents (Figure 11a,b, respectively) shows that the size of the NCs increases with increasing S concentration. Therefore, the broadening of that reflection is not associated with size effects. Thus, we interpret the anisotropic broadening of one reflection (compared to that of the others) as a decrease in the coherence length of the respective plane. This, together with the increasing size of the NCs with S content, points toward the existence of planar defects. Thus, both our TEM and XRD results confirm the presence of a high degree of stacking faults within the particles with increasing S content in the reaction mixture. The Cd atoms in both the WZ and ZB local environments are tetrahedrally coordinated. Differences in their NMR chemical shift arise due to changes in bond angles and lengths between the Cd atom and its neighbors in higher coordination shells, reflecting differences in the average local atom-pair correlations of Cd and its neighbors. We expect that Cd atoms in the vicinity of stacking faults would experience slight differences in their chemical and electronic environments compared with the pure phases or the ZB and WZ-like local environments. These would result in a distribution of chemical shifts that is observed in our RFDR spectra. While at this stage we cannot assign the specific resonance frequencies detected to unique chemical environments, this can in principle be done by ab initio quantum mechanical calculations of the NMR shifts for different structural models taking into account the presence of stacking faults. Thus, the high sensitivity from MIDNP provides an effective route for detecting structural defects in the bulk of NCs that could be used to rationalize the effect of different synthetic conditions (here, S content) on the resulting particles.

## 3 Conclusions

We performed a detailed investigation of Mn(II) doping in CdS NCs toward implementation of functional Mn(II) dopants as DNP polarizing agents. The presence of strong isotropic couplings between Mn(II) and Cd nuclei detected by EPR hyperfine spectroscopy confirmed incorporation in the NCs. Thorough TEM investigation, supported by simulating surface vs bulk doping scenarios, confirmed predominantly bulk doping and allowed us to quantify the dopant distribution across the particles.

We demonstrated that Mn(II) doping provides a viable route for sensitivity enhancement through MAS-DNP of semiconducting CdS NCs, providing one order of magnitude increase in sensitivity. We found that the synthetic conditions have a strong effect on DNP enhancement, with higher sensitivity obtained for low Mn(II) doping as well as a longer particle incubation time.

The sensitivity gained by MIDNP enabled probing of both surface and core atomic environments of CdS NCs. Furthermore, with the increased sensitivity in 2D, correlation experiments could be efficiently performed, revealing the presence of structural defects in the bulk of the NCs. These defects are likely associated with the presence of stacking faults in CdS NCs as observed in high-resolution TEM.

Our results show that MIDNP is a viable approach for increasing the sensitivity of ssNMR with minimal chemical interference. Ideally, one can make use of the same dopants that are needed for the NC functionality to increase the NMR sensitivity for characterization. While this scenario is not a general one, we do expect that Mn(II) dopants, which are often introduced in NCs for tuning their optical and electronic properties, can also be utilized as “structural spies” as they are typically viable for MIDNP. Additionally, the applicability of this method can be extended to systems where the dopants are not necessarily introduced for property enhancement. In such cases, the desired dopant should reside in substitutional sites, where only strain is expected to be the main deformation in order to minimize defect formation.

While limited in absolute sensitivity gains compared to the exogenous DNP approach, in partucular for surface sites, MIDNP offers unique advantages for investigating the NC–ligand interactions as well as detecting a low population of defects in their bulk. Despite these limitations, we expect the presented methodology to be a valuable tool in the arsenal of approaches used to rationally design functional NCs, especially in cases where the doping strategy also enhances the material’s performance.

## 4 Experimental Section

### 4.1 Materials

Cadmium chloride (CdCl_2,_ >95%), sulfur (S, >99.5%), and tri-octyl-phosphine oxide (99%) were purchased from Acros Organics. Manganese acetate tetrahydrate (C_4_H_6_MnO_4_-4H_2_O, 99.999%) and octylamine (99%) were purchased from Alfa Aesar. Hexane (>95%) was purchased from Gadot Chemical. All chemicals were used as received without further purification.

Synthesis: In a three-necked flask fitted with a condenser and a thermometer, 183 mg of CdCl_2_ (1 mmol) was added to 20 mL of octylamine. The flask was connected to a Schlenk line and purged with nitrogen gas. In a separate vial, 51.2 mg (1.6 mmol) of elemental sulfur was added to 5 mL of octylamine. The solution was mixed using a vortex until the complete dissolution of sulfur, resulting in a transparent yellowish-red solution. The metal precursor solution was heated to 180 °C until the metal salts were completely dissolved. The sulfur solution was transferred to a syringe and injected into the heated metal precursor. The reaction was initiated and allowed to proceed for a predetermined duration (10 min to 72 h). It was then quenched by cooling in an ice bath, followed by the addition of 25 mL of hexane to the flask. The content of the flask was transferred to a 50 mL test tube for centrifugation. The slurry was taken out of the centrifuge, washed with hexane, and centrifuged again. Doping was carried out by introducing Mn acetate into the metal precursor solution at varying concentrations.

### 4.2 DNP-NMR

Samples for MAS-DNP experiments were prepared under an inert atmosphere as follows. NCs were crushed to fine powders using a pestle and mortar and transferred to a 3.2 mm sapphire rotor sealed with a Teflon insert and a ZrO_2_ cap. The rotors were initially spun at RT before introducing them to the cooled DNP probe. ssNMR experiments were performed on 9.4 T Bruker Avance III and Avance Neo 400 MHz wide-bore spectrometers equipped with a sweeping coil and a 263 GHz gyrotron system. A 3.2 mm double-resonance low-temperature DNP probe was used for the experiments at a MAS frequency of 9 kHz. Direct excitation experiments were performed with single pulse excitation with a radio frequency amplitude of 108 kHz. RFDR experiments were performed with 100 increments in the indirect dimension, a polarization delay of 60 s, and 16 scans, corresponding to a total experiment time of 27 h. Polarization build-up times with microwave irradiation, *T*_bu_, were measured with the saturation recovery pulse sequence using a train (50 repetitions) of short pulses separated by 1 ms for saturation. The saturation recovery pulse sequence was used to measure DNP build-up times. The obtained build-up curves were fitted to a stretched exponential function according to
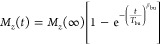
where *T*_1,bu_ is the longitudinal relaxation or build-up time under microwave irradiation and β_1,bu_ is the corresponding stretched exponent factor.

The signal intensity for calculating enhancement factors, ε_on/off_, was determined by integration over the entire range of resonances in TOPSPIN. DMFIT was used for the spectral deconvolution of resonances.^58^ The ^113^Cd signal was referenced to commercial CdS powder at 65 ppm, purchased from Holland Moran.^17^ The corresponding XRD pattern from that sample verifies that the dominating phase is CdS-ZB (see Figures S4 and S5).

### 4.3 EPR Experiments

CW EPR measurements were carried out on a Bruker Magnettech ESR5000 spectrometer operating at 9.46 GHz. Microwave saturation experiments were carried out between 0.1 and 100 mW. Unless stated otherwise in the text, measurements were carried out at temperatures of 100 K. Pulse EPR measurements were performed on a Bruker Elexsys E580 spectrometer operating at 9.8 GHz with an X-band resonator ER4102ST. Q-band measurements were carried out at 35 GHz and fitted with a Q-band resonator (EN-5107-D2). The temperature was controlled by an Oxford Instruments CF935 continuous-flow cryostat by using liquid He. All measurements were carried out at a temperature of 15 K.

ESEEM three-pulse sequences (π/2−τ–π/2–T−π/2–echo) were carried out in a magnetic field where the echo intensity is maximum, and the lengths of the π/2 and π microwave pulses were 10 and 20 ns, respectively. The pulse interval T was set to 151 ns to maximize the ^113^Cd modulation according to , where q is an integer and ν_I_ is the ^113^Cd nuclear Larmor frequency.^68^

The ENDOR spectrum was measured using the Davies ENDOR pulse sequence, π–T−π/2−τ–π–τ–echo, with the RF pulse applied during the time interval *T*. The experimental conditions were as follows: *t*_π_ = 200, *t*_π/2_ = 100, τ = 500 ns, and the RF pulse length *T* was 12 μs.

EPR spectra simulation and fitting were carried out using the EasySpin 6.0.0 and EasySpin 5.2.35 packages.^41^

### 4.4 Electron Microscopy

Samples were prepared for TEM by drop-casting a suspension of CdS NC in hexane onto a lacey carbon Cu grid. Bright-field and high-angle annular dark-field (HAADF) STEM images were taken using a Talos FX200 field emission electron gun microscope operating at 200 kV. High-resolution STEM micrographs were taken utilizing a probe-aberration corrected FEI Themis Z operated at 200 kV. EDS hyperspectral data were obtained with a Super-X G2 four-segment SDD detector with a probe semi-convergence angle of 21.4 mrad and a beam current of approximately 1.05 nA. The EDS data cubes were initially processed with the Velox software for background subtraction.

The STEM micrograph simulation was carried out utilizing MATLAB coding. The mean radius and standard deviation were taken from the experimental data to simulate 3D spheres with the same values within a 3D slab. Each voxel in the simulated micrographs was assigned a volume equal to the cube of the pixel size in the experimental STEM data. Cd atoms in each voxel in the sphere were calculated and modified according to the type of simulation used. Eventually, the final pixel intensity in the resulting image was obtained by summing every plane in the 3D slab. The images were used as—is to simulate the bulk EDS signal. Applying the 3D Sobel operator generates a matrix containing the surface atoms, resulting in surface simulation. In each case, we achieved the corresponding simulation from the dopants by partially substituting the Cd atoms with Mn atoms.

The quantitative STEM analysis was carried out using open-source software. Specifically, we used Fiji^69^ and Cellpose.^70^ The out-of-the-box pretrained Cellpose model was used to identify the Cd particles in the STEM-HAADF image. Fiji’s distance transform plugin was then used to calculate the distance of the Mn atoms from the particle edge. Only Mn signal intensity above a fixed threshold was considered, assuming that for dopant counting if pixel intensity is above that threshold, it corresponds to a single dopant atom. The output of the Fiji macro is the summation of signal intensity as a function of its distance from the particle edge. The Fiji macro and MATLAB scripts are deposited on GitHub and available for download (https://github.com/WIS-MICC-CellObservatory/Metal-ions-in-nanocrystals/).

Fiji can be downloaded from https://imagej.net/software/fiji/downloads; Cellpose can be downloaded from https://github.com/mouseland/Cellpose; and MATLAB is available at https://www.mathworks.com. For dopant counting, we assumed that if the pixel intensity is above the threshold, then that will correspond to a single dopant atom.

### 4.5 XRD

XRD measurements were performed on an Ultima-III Rigaku diffractometer. The X-ray (Cu Kα radiation) tube voltages and currents were 40 kV and 40 mA, respectively. The measurement range of 2θ was from 10° to 70°, with a scan rate of 1° per minute. Powder XRD measurements on CdS NC were performed in reflection geometry using Rigaku (Tokyo, Japan) theta–theta diffractometers: an Ultima-III equipped with a sealed copper anode tube operating at 40 kV/40 mA.

### 4.6 Inductively Coupled Plasma Mass Spectrometry

ICP-MS measurements were carried out on Agilent 7700s ICP-MS system for ultratrace (ppb) elemental analysis applications. It features high sensitivity and a wide range of interference removal technologies with an ORS3 collision/reaction cell. The samples are introduced into the instrument via an autosampler system.

## Supporting Information Available

Additional characterization results including electron microscopy images, solid-state NMR spectra, and XRD, as well as electron spin relaxation times estimated by fitting EPR saturation curves (PDF)
