# Supplementary material for: Metal Ion Dynamic Nuclear Polarization in Mn(II)-Doped CdS Nanocrystals: Atomic-Scale Investigation of the Dopant and Its Host
Source: ACS Nano. 2025 Apr 28;19(18):17640–52. doi: 10.1021/acsnano.5c01257 (PMC12080374; doi:10.1021/acsnano.5c01257)
Supplement: Supplementary file 1 — nn5c01257_si_001.pdf [file nn5c01257_si_001.pdf]

# Supporting Information

## Metal Ions Dynamic Nuclear Polarization in Mn(II) Doped CdS Nanocrystals: Atomic Scale Investigation of the Dopant and its Host

Ran Eitan Abutbul[†], Daniel Jardon-Alvarez[†], Lothar Houben[‡], Ofra Golani[#], Ehud Sivan[#], Raanan Carmieli[‡], Ilia Kaminker[§], Michal Leskes[†*]

[†]Department of Molecular Chemistry and Materials Science, Weizmann Institute of Science, Rehovot, 761000, Israel

[‡]Department of Chemical Research Support, Weizmann Institute of Science, Rehovot, 761000, Israel

[#]Department of Life Sciences Core Facilities, Weizmann Institute of Science, Rehovot, 761000, Israel

[§]School of Chemistry, Faculty of Exact Sciences, Tel Aviv University, Tel Aviv 69978, Israel

*michal.leskes@weizmann.ac.il

**Table S1**. $T_{1e}$ calculated by fitting the corresponding EPR saturation curves from CdS NCs synthesized at different Mn concentrations.

| Mn [mM] solution | $T_{1e}$ |
|---|---|
| 56 mM | 3 µs |
| 169 mM | 1 µs |
| 270 mM | 0.5 µs |

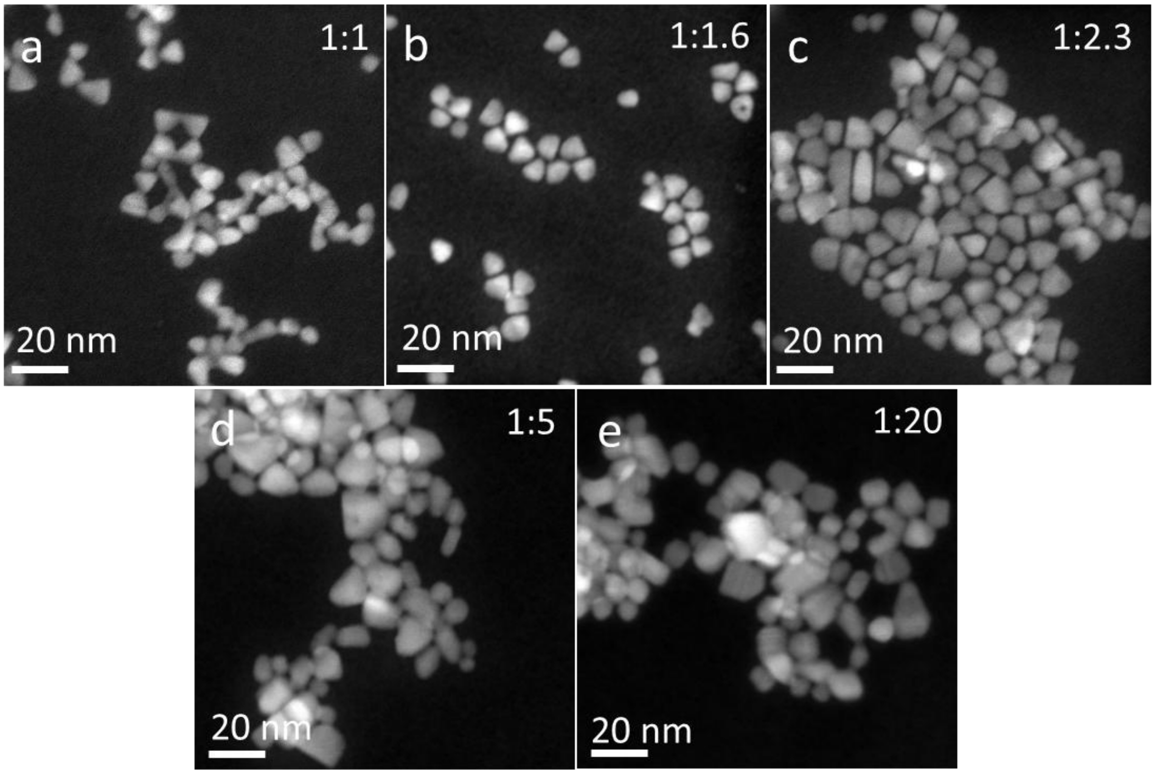


**Figure S1**. STEM micrographs of Mn-doped CdS NCs synthesized with various Cd:S ratios (a) 1:1 (b) 1:1.6 (c) 1:2.3 (d) 1:5 (e) 1:20

**Table S2**. $T_{1e}$ calculated by fitting the corresponding EPR saturation curves from CdS NCs synthesized at different Cd/S ratios.

| Cd/S ratio | $T_{1e}$ |
|---|---|
| 1:1.6 | 1.5 μs |
| 1:2.3 | 0.6 μs |
| 1:5 | 0.3 μs |
| 1:20 | 0.2 μs |

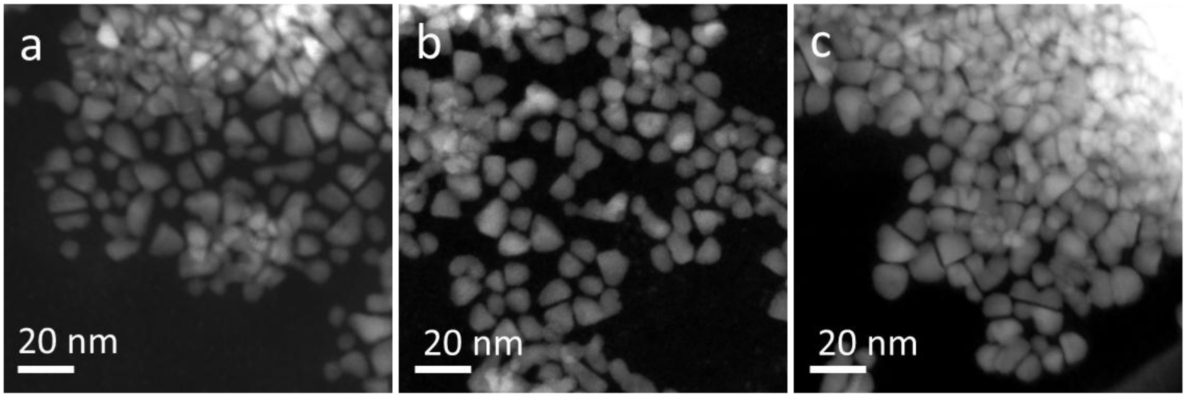


**Figure S2**. STEM micrographs of CdS NCs syntheized in the presence of (a) 0.07mM (b) 0.2 (c) 0.3 of Mn at incubation time of 24 h.

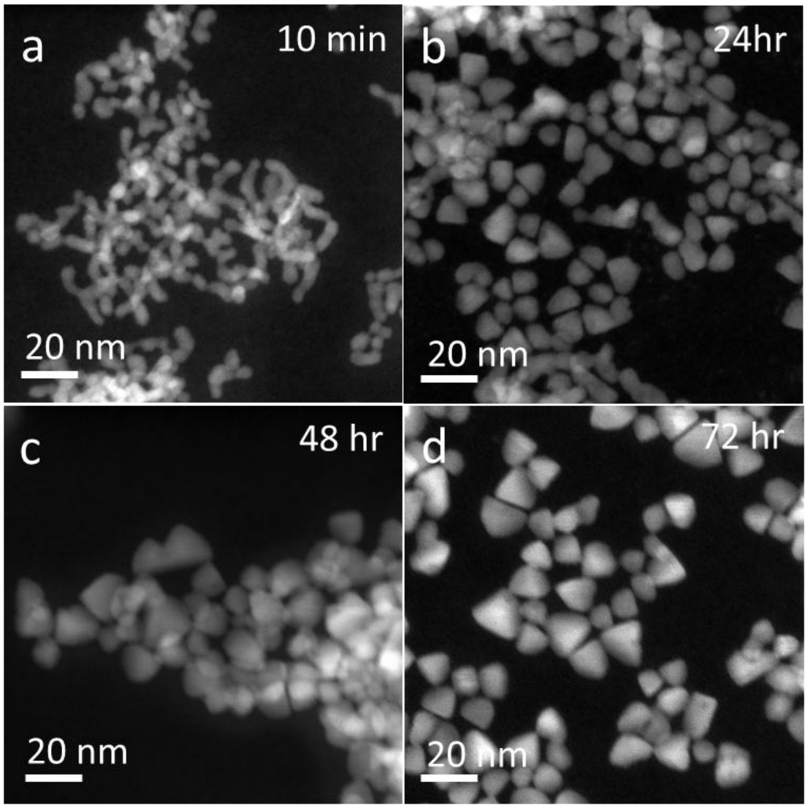


**Figure S3**. STEM micrographs of CdS NCs syntheized in the presence of 0.2mM of Mn with different incubation times. (a) 10 min (b) 24 hr (c) 48 hr (d) 72 hr .

**Table S3**. $T_{1e}$ calculated by fitting the corresponding EPR saturation curves from CdS NCs synthesized at different incubation times.

| Growth time | $T_{1e}$ |
|---|---|
| 10 min | 0.33 μs |
| 24hr | 1 μs |
| 48hr | 1.02 μs |
| 72hr | 0.6 μs |

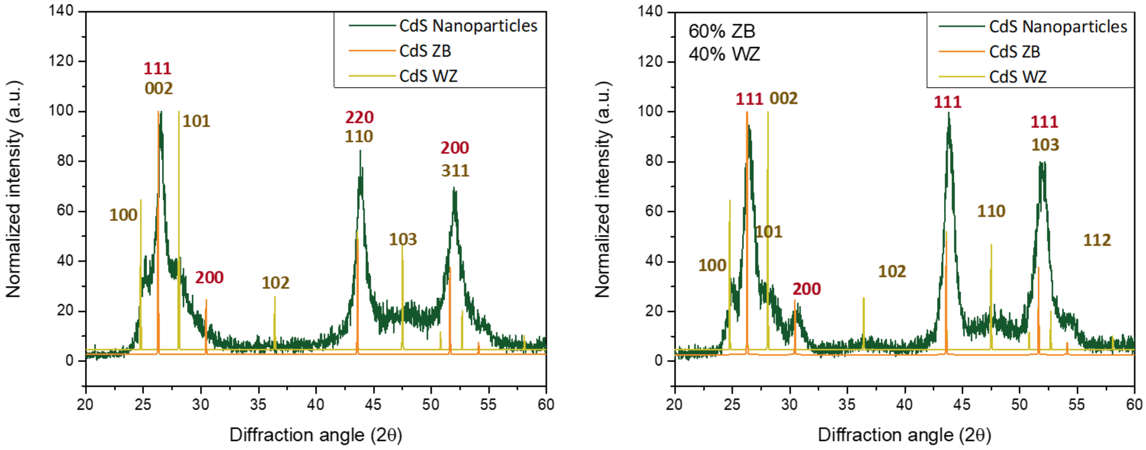


**Figure S4**. XRD diffractograms of (a) CdS purchased from Holland-Moran (b) as-synthesized CdS NCs. 0.2 mM of Mn in solution, grown for 24h.

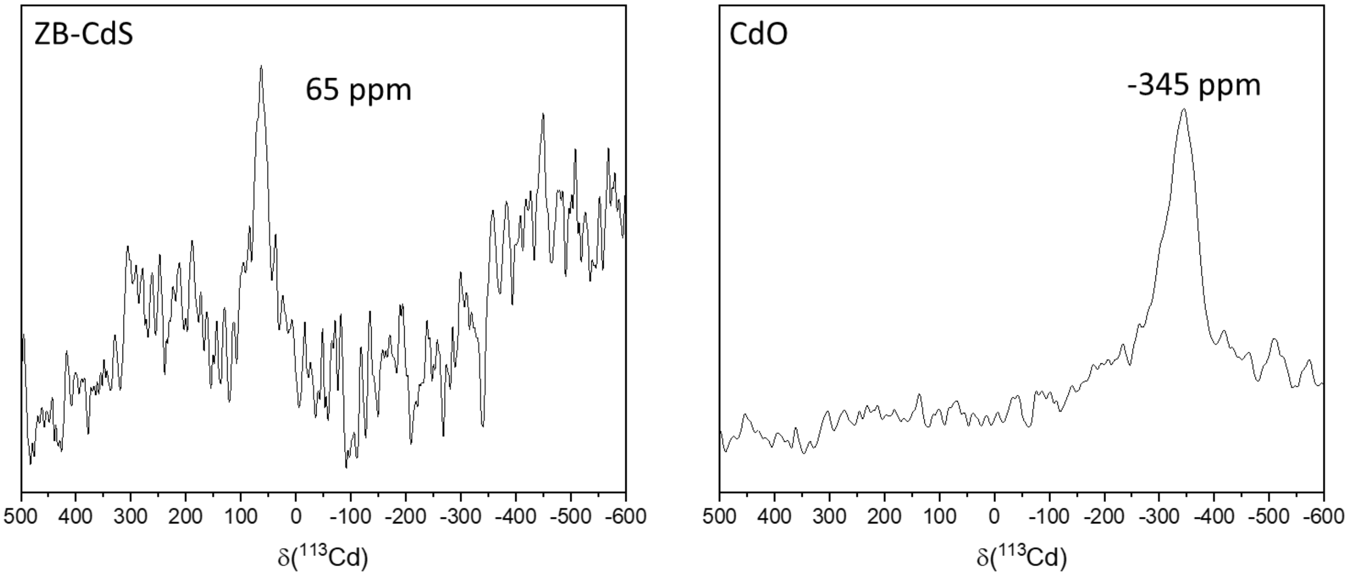


**Figure S5**. $^{113}$Cd NMR reference spectra of CdS and CdO purchased from Holland-Moran.

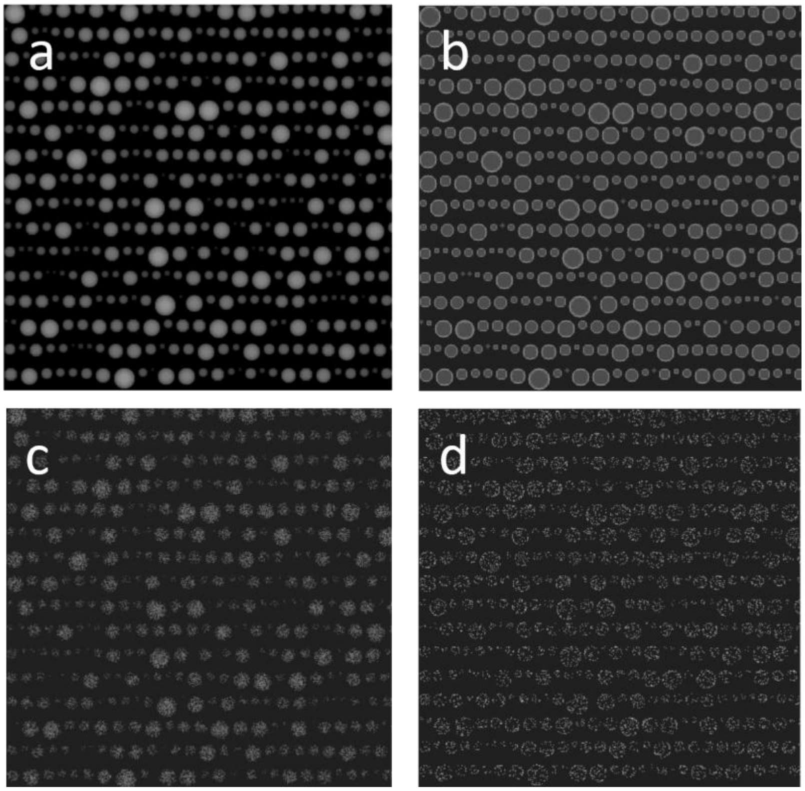


**Figure S6** Simulated elemental maps of spherical particles with similar size and shape distribution as the studied CdS NCs. (a) Cd and (c) Mn maps where the ions occupy the volume in the core and surface of the NCs. (b) Cd and (d) Mn maps where the ions occupy only the volume on the surface of the NCs. An Mn/Cd ratio of 1/50 was used in both cases.

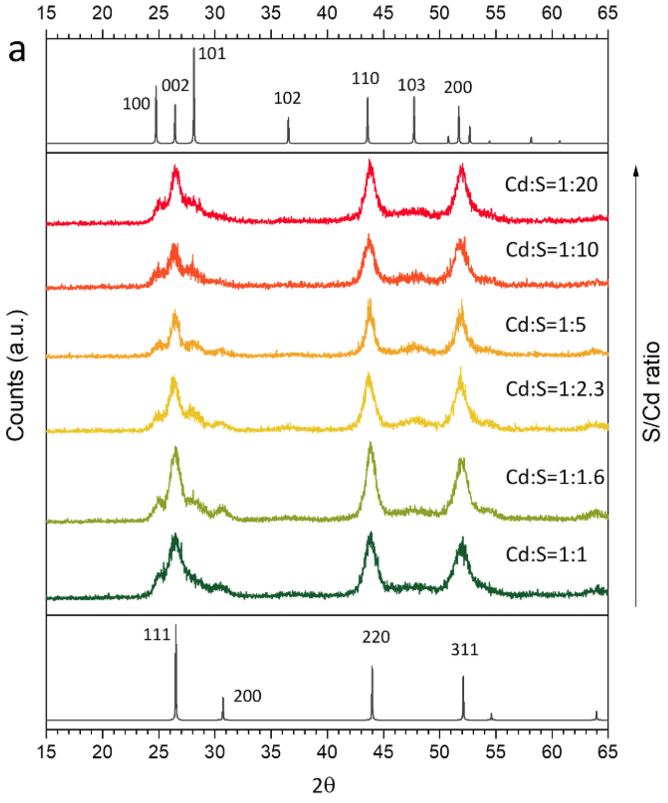


**Figure S7**. a. XRD diffraction patterns taken from samples synthesized with various S/Cd ratios. from 1 to 20.
